# Notes and News

**Published:** 1900-11-10

**Authors:** 


### NOTES AND NEWS.

One of the new wards at the Boscombe Hospital, Bournemouth, has, by permission of the Queen, been named the Empress Victoria Ward, and the hospital will hereafter bear the title of "Royal" Institution. The new additions to the hospital have cost £10,000.

A new isolation hospital has been opened at Basingstoke. The cost has been £5,555.

The South African Hospitals Commission has resumed its enquiry at Burlington House. Mrs. Richard Chamberlain was the first witness called before the Commissioners on the present occasion.

Lord Rothschild has issued an appeal on behalf of the Throat Hospital in Golden Square. We commend the appeal to hospital managers for the effective excellence of its form.

The seaman who was stricken with the plague on the *Marienburg* on its voyage from Buenos Ayres has died at Bremen. The patient was discharged from the ship before it was discovered that he was suffering from the plague, but so far no one who associated with him has shown any signs of contracting the disease. The *Marienburg* is detained in quarantine at Antwerp.

The foundation stone of one of the first consumptive sanatoria to be carried out by a company on a paying basis has just been laid in Kinross-shire, at Ochils. The capital subscribed is £35,000. Ochils stands 800 feet above the sea level, and the site is sheltered by wooded country, with a frontage to the south. Fifty patients are to be provided for. Kinross records the lowest death rate from pulmonary affections in the British Isles.

Both King's College and the Welsh Hospital in South Africa have sustained a loss in the death of Professor A. Hughes. Professor Hughes, who was Professor of Anatomy at King's College and originator and chief organiser of the Welsh Hospital for South Africa, arrived home on October 19th in the *Saxon*, and died on Saturday last of enteric fever developed two days after saling from Cape Town. Professor Hughes went out in June to join the hospital owing to the large amount of sickness which had occurred amongst the staff. The Welsh Hospital has done excellent work, shown by the fact that Lord Roberts has asked to have its assistance for a further three months.

Within three months the Italian Hospital has been so unfortunate as to lose both patron and president in the persons of the late King Humbert and Baron de Renzis, Italy's Ambassador to Great Britain.

A new home for incurables for Yorkshire is to be erected at Harrogate adjoining the Royal Bath Hospital. Sir Christopher Furness has already given £200 towards the project, and has promised further help if necessary. The cost is estimated at £15,000.

At the Ipswich meeting of the British Medical Association, a committee was appointed to consider and report upon the best means of reorganising the constitution of the British Medical Association, and was instructed to furnish a provisional report to the branches by February 1, 1901. Mr. Edmund Owen is the chairman. The committee is stated to have already made considerable progress in establishing certain principles upon which the new constitution should be based.

The Leeds Workpeople's Hospital Fund is an active organisation. It maintains two convalescent homes, one for men and one for women, and collected nearly £4,500 during the past year. At the last quarterly meeting a proposal was put forward for the provision of a new hospital on the south side of the city. The General Infirmary can now hardly cope with the calls upon its accommodation. If a new hospital were the outcome of the Workpeople's Hospital Fund it would be a splendid testimony of the intelligent interest taken by the people of Leeds in movements for their benefit, and a proof that the work of the Infirmary is deservedly appreciated.

Ladies pursuing medical studies in Vienna are undergoing much the same difficulties as beset Mrs. Garrett Anderson and her few pioneer colleagues at Edinburgh earlier in the century. Both students and lecturers are offering opposition to the new academic law of admission for female students. It is reported of one professor that he refused to continue his discourse whilst ladies occupied the front row of seats at his lecture. Whether he would have continued had they occupied a less conspicuous position we are not told, but we should have imagined that the ladies themselves would have preferred to be seated behind rather than in front of the male students. The latest development is the decision on the part of the mothers to attend their daughters to lectures in the hope of screening them from the insults and annoyance they are subjected to.

To commemorate the anthropological work of the late Professor Huxley, the Council of the Anthropological Institute of Great Britain and Ireland has decided to found a public lecture, which will be called the "Huxley Memorial Lecture," and will be given annually at the opening of the winter session of the Institute. The first Huxley Lecture will be delivered by the Right Hon. Lord Avebury, D.C.L., LL.D., F.R.S., and is announced to take place on Tuesday, November 13th, at 8.30 p.m., in the Lecture Theatre of the Museum of Practical Geology, in Jermyn Street, S.W., which was the scene of much of Huxley's teaching. Lord Avebury will take as the subject of his discourse Huxley's own contributions to anthropological science. Applications for tickets of admission should be addressed to the Secretary, the Anthropological Institute, 3 Hanover Square, W.

